# Isolation and functional characterization of novel biosurfactant produced by *Enterococcus faecium*

**DOI:** 10.1186/2193-1801-4-4

**Published:** 2015-01-07

**Authors:** Deepansh Sharma, Baljeet Singh Saharan, Nikhil Chauhan, Suresh Procha, Sohan Lal

**Affiliations:** 9 Department of Microbiology, Kurukshetra University, Kurukshetra, 136 119 INDIA; 10 Dairy Microbiology Division, National Dairy Research Institute, Karnal, Haryana 132 001 India; 11 Division of Microbiology and Immunology, Vector Control Research Center, Puducherry, 605006 India; 12 Department of Chemistry, Kurukshetra University, Kurukshetra, 136 119 India

**Keywords:** Biosurfactant, Glycolipids, Probiotic, Lactic acid bacteria, Cytotoxicity

## Abstract

The objective of the present study was to isolate the biosurfactant (BS) producing lactic acid bacteria (LAB) from traditional fermented food (buttermilk) and its functional and structural characterization. BS isolated from strain MRTL9 reduced surface tension from 72.0 to 40.2 mN m^-1^. The critical micelle concentration (CMC) of BS was 2.25 mg ml^-1^ with emulsification efficiency (E_24_) after 24 h of 64% against kerosene oil. The cell bound BS was partially purified by silica gel column chromatography and found as glycolipid. The gas chromatography and mass spectroscopy data revealed the fatty acid as hexadecanoic acid. Xylose was determined as hydrophilic moiety. The BS was found to be stable to pH changes over a range of 4.0-12.0, being most effective at pH 7 and showed no apparent loss of surface tension and emulsification efficiency after heat treatment at 120°C for 15 min. The outcomes of cellular toxicity showed lower toxicity of BS in comparison to SDS and rhamnolipids. Current study confirmed the preventive anti-adhesion activity of BS. These amphiphilic molecules, interferes with the microbial adhesion and found to be least cytotoxic with cellular compatibility with mouse fibroblasts cells.

## Introduction

Microbial surfactants are amphiphilic molecules which exhibit high surface activity and emulsifying properties. Microbial surfactants can be potential substitutes to chemical surfactants due to their stability under extreme conditions, structural diversity, low toxicity and biodegradability. BS are commonly employed in crude oil recovery (Banat et al. 2010) hydrocarbon degradation in soil (Nitschke et al. 2011; Saharan et al. 2011; Sharma and Singh 2014; Saharan et al. 2014) heavy metals removal from contaminated soil (Juwarkar et al. 2007) and hydrocarbon biodegradation in aquatic environment (Khire 2010). Surface interactions are mediated by the amphiphilic nature of the molecules consists of hydrophilic regions (acid, peptide cations, or anions, mono-, di- or polysaccharides) and hydrophobic regions (unsaturated or saturated hydrocarbon chains or fatty acids), which allow them to act as surfactants at the interfaces (Banat et al. 2010; Myers 2005). The availability of commercially produced BS are limited to surfactin, sophorolipids and rhamnolipids (Saharan et al. 2011). Lactobacillus spp. are potent BS producing microorganism predominately found in the gastrointestinal microflora of human and animals. BS derived from lactic acid bacteria contributes to their high attributes of prevention of bacterial infections in the human body (Gudina et al. 2011). A number of studies reported the potential of lactobacilli as BS producers and their significant role in public health (Velraeds et al. 1996; Heinemann et al. 2000; Rodrigues et al. 2004; Servin 2004; Rodrigues et al. 2006a, b; Falagas and Makris 2009; Augustine et al. 2010; Saravanakumari and Mani 2010; Thavasi et al. 2011; Gudina et al. 2011; Saharan et al. 2011; Rodriguez-Pazo et al. 2013; Moldes et al. 2013). Silicone rubber material is extensively used in sophisticated medical instruments and voice prostheses due to its ease of casting and exceptional mechanical properties. But the hydrophobic silicone rubber surfaces becomes colonized with biofilm forming pathogens including bacterial and yeast strains (Monteiro et al. 2011). Hydrophobic silicone rubber surface becomes colonized by a variety of bacterial and yeast strains (Neu et al. 1990). Laryngectomized patient encountered problems associated with biofilms formation on silicone rubber surfaces. Biofilm formation associated with severe problems once the biofilm formation has prolonged. Blocking and leakage due to biofilm formation affect the function of the device and need to be change regularly (Busscher and Van der Mei 1997). Application of BS to a surface modifies its hydrophobicity, interferes microbial adhesion and desorption processes; in that sense, the production of BS by probiotic bacteria *in vivo* can be considered as a defense against other colonizing food borne pathogens (Van Hoogmoed et al. 2000; Nitschke et al. 2009). BS coating decreases the contact angle of silicone surface and it becomes hydrophilic (Rodrigues et al. 2006a). BS derived by lactic acid bacteria impaired biofilm formations on silicone rubber and other biomedical instruments surfaces (Velraeds et al. 1996; Busscher et al. 1997; Velraeds et al. 1997; Velraeds et al. 2000; Van der Mei et al. 2000; Rodrigues et al. 2004; Walencka et al. 2008; Fracchia et al. 2010; Gudina et al. 2011). The present work describes the production of novel BS produced by LAB isolates from butter milk. The current study also includes functional, structural and cytotoxic characterization of the BS produced. As far as literature surveyed, this is the first compilation of data on BS production from *E. faecium* and its structural attributes.

## Materials and methods

### Isolation of LAB

BS producing LAB was isolated from indigenous buttermilk sample by enrichment in 100 ml of sterile minimal media (MM) in Erlenmeyer flask with 1% paraffin oil as carbon source. The suspension was incubated at 37°C for 48 h. After incubation the inoculum from the culture flasks was sub cultured on deMan Rogosa and Sharpe (MRS) agar medium contains (20 g D-Glucose l^-1^, 10 g Peptone l^-1^, 10 g Beef extract l^-1^, 5 g Yeast extract l^-1^, 2 g Di-potassium phosphate l^-1^, 2 g Tri-ammonium hydrogen citrate l^-1^, 5 g Sodium acetate l^-1^, 0.2 g Magnesium sulphate l^-1^, Manganese sulphate l^-1^ and 1 g Tween-80 l^-1^). LAB were stored at -20°C in MRS broth containing 20% (v/v) glycerol stock as master stock until they were used in current study.

### Screening for BS production

BS production was examined with various methods *i.e.* drop collapse (Morikawa et al. 1993), emulsification index (Youssef et al. 2004) and surface tension (ST) measurement after 72 h of growth.

### Measurement of surface tension and CMC determination

ST is an indicator of washability, wetting, and other surface related properties of any surfactant. ST of supernatant was measured by the De Nouy ring method, using a tensiometer equipped with a 1.9 cm platinum ring at room temperature (Lauda, Germany). ST of cell-bound biosurfactant was determined by measuring directly the ST of cell bound biosurfactant. Briefly, LAB cells were recovered at the end of experiment by centrifugation (10,000 g, 10 min, and 4°C), washed twice in demineralized water and resuspended in Phosphate buffer saline (PBS, pH 7.2) with gentle stirring for 2 h. The sterile PBS was used as a control. The Instrument was calibrated twice by measuring ST of distilled water. The critical micelle concentration (CMC) was also determined by measuring the surface tension of serially diluted BS. The BS solution was prepared and diluted using Tris–HCl buffer, pH 8 (Faria et al. 2011). All the measurements were observed in triplicates.

### Taxonomic identification

#### Colony PCR

Colony PCR was performed for taxonomic identification of the LAB isolate (Sheu et al. 2000). Briefly, the optimized colony PCR reaction mixture contained 1X PCR amplification buffer (20 mM (NH_4_)_2_SO_4_, 72.5 mM Tris/HCl, 0.1% Tween 20, pH 9.0), 2.5 mM MgCl_2_, 200 μM of each deoxynucleotide triphosphate, 2.5 μM of each primer 27f (5-AGAGTTTGATCMTGGCTCAG-3) and 1385r (3-AATTCAAATTTAATTTCTTTCC-5), 1.25 U DNA polymerase in 50 μl PCR reaction mixture. Approximately 1 mm diameter colonies were picked up with sterilized toothpick and directly transferred to the PCR tube as DNA templates. The thermal cycle programme, run on a thermocycler PCR system (Eppendorf, Germany) consisted of one cycle of 94°C for 10 min, 51°C for 2 min, 72°C for 2 min, and 35 cycles of 94°C for 20 s, 57°C for 45 s (decreased by 1 s per cycle), 72°C for 1 min, and then incubation at 72°C for 5 min, and a final incubation at 4°C. PCR-amplified DNA fragments were observed on 1.3% agarose gel in TAE buffer (0.04 M Tris acetate, 0.02 M acetic acid, 0.001 M EDTA), containing 1 g/ml of SYBR green. Briefly, 10 μl of each amplification mixture and the molecular mass marker were separated on agarose gel electrophoresis. The amplified DNA fragments were visualized by UV illumination. The 16S rRNA gene sequence obtained from the isolate was compared with other bacterial sequences by using NCBI mega BLAST (http://blast.ncbi.nlm.nih.gov/Blast.cgi) for their pair wise identities. Multiple alignments of these sequences were carried out by Clustal W of EBI (http://www.ebi.ac.uk/cgi-bin/clustalw/) with 0.5 transition weight. Nucleotide composition of each aligned sequence was determined by DNA baser software package (http://www.dnabaser.com/) and the 16S rRNA sequence was deposited in GenBank.

### Production media and cultivation conditions

For BS production in shake flask condition, 250 ml of MRS-Lac (glucose was replaced by lactose) broth (pH 6.2) was inoculated with 1% (v/v) of pre-culture and incubated for 72 h at 37°C). After 72 h, cells were harvested by centrifugation (10,000 g, 10 min, 4°C), washed twice and resuspended in 100 ml of PBS (Rodrigues et al. 2006b). The suspension was kept at room temperature for overnight with gentle stirring for the release of cell-bound BS. Cell free suspension was filtered through a 0.22 μm pore size filter (Axiva, India). The suspension was dialyzed against demineralized water at 4°C in a dialysis membrane (molecular weight cutoff 6,000-8,000 Dalton, Himedia, India) and freeze dried.

### Purification of BS

The cell-bound BS was partially purified in silica gel (60–120 mesh) column eluted with gradient of chloroform and methanol ranging from 20:1 to 2:1 (v/v). The fractions were pooled after TLC analysis and solvent was evaporated.

### Structural characterization

#### Thin layer chromatography (TLC)

The composition of the cell-bound partially purified BS was determined by TLC followed by post chromatographic detection. Briefly, 4 ml of PBS extract was extracted twice with ethyl acetate 1:1.25 (Rankem, India). Upper phase was extracted two times with ethyl acetate and the ethyl acetate was allowed to evaporate at room temperature (Syldatk et al. 1985; Schenk et al. 1995; Hormann et al. 2010). 1 ml aliquot of BS extract was concentrated and separated on a precoated silica gel plate (Merck, India) using chloroform/methanol/glacial acetic acid (65:15:2 v/v) as mobile phase. The polysaccharides moities were stained with Syldatk reagents (anisaldehyde: sulfuric acid: glacial acetic acid 0.5:1:50), whereas the fatty acid moieties were stained with ammonium molybdate/cerium sulfate (0.42%, w/v; ammonium molybdate and 0.2%, w/v, cerium (IV) sulfate in 6.2% sulfuric acid). Plates were heated at 120°C for 10 min. The chromatograms of the BS were compared with the TLC pattern of a standard mixture of di-rhamnolipids of *Pseudomonas aeruginosa* which was prepared from Jeneil JBR 425 (Jeneil Biosurfactants Company, Saukville, United States).

### Ionic property of BS

The ionic property of cell bound BS was determined by using agar well diffusion method (Meylheuc et al. 2001). Briefly, 3 uniformly spaced wells were made on a soft agar (1%) plate, central well was filled with 10 μl of BS. Either sides of wells were filled with anionic compound (Sodium dodecyl sulfate, 20 mM) and cationic compound (Cetyl trimethyl ammonium bromide (CTAB), 20 mM). Plates were incubated at 25°C for 24 h and observed for the precipitation lines.

### Fourier transform infrared spectroscopy (FTIR) and Nuclear magnetic resonance (NMR) spectroscopy

Molecular components of partially purified cell bound BS was elucidated using FTIR spectroscopy by scanning it in the range of 4000–400 cm^-1^ at a resolution of 4 cm^-1^ with resolution of 2 wavenumbers per wavenumber. (Model-ABB & MB-3000). The 1–3 mg BS was dissolved in 100% CDCl_3_ and ^13^CNMR analysis was carried out using a Bruker Av II-400 spectrometer. Both proton and carbon NMR chemical shifts were stated in ppm relative to the solvent shift as chemical standard. Peaks were compared and predicted with the data reported previously.

### Liquid chromatography (UPLC) and mass spectroscopy

Aliquots of the partially purified BS was dissolved in methanol to obtain 1 mg ml^-1^ solution. Waters (UPLC) system equipped with quaternary gradient pump, auto sampler and a Photo diode detector (PDA, 2996) were used in present study. Separation was performed with C18 column (1.7 μm × 2.1 μm × 100 mm) at column oven temperature 40°C. A multistep linear gradient composed of eluent A (Water + 0.1% trifluoroacetic acid) and eluent B (Acetonitrile + 0.1% trifluoroacetic acid) was applied. The auto sampler temperature was maintained at 10°C. 10 μl of sample solution was injected. From 0–13 min a linear gradient was applied from the mixture A: B (70:30, v/v) to A: B (0: 100 v/v). A plateau of 100% eluent B from 13 min to 15 min was set before going back to 70% eluent A from 15 min to 16 min. Flow rate of mobile phase was 0.3 ml/min. The LC system was coupled with a Waters mass spectrometer with an atmospheric pressure electroscopy interface. The ESI source was set in positive and negative ionization mode. Nitrogen gas was used as nebulizer gas and Helium gas a collision gas (Janek et al. 2013).

### Gas chromatography and mass spectroscopy (GC-MS)

#### Fatty acids analysis

The sample was reconfirmed on gas chromatograph-mass spectrometer system equipped with a VF-5MS column (Thermo Scientific TSQ 8000). Briefly, the initial column temperature was 100°C for 1 min, then ramped at 30°C min^-1^ to 270°C, and finally held at 270°C for 10 min. The temperatures of the transfer line, ion trap, and quadrupole were 280, 230, and 150°C, respectively. The inlet temperature was 270°C, and a 100 μL sample was injected. The flow rate of the carrier gas (helium) was 1.0 mL min^-1^.

#### Thermal gravimetric (TG) analysis

TG analyses of freeze dried partially purified BS were carried out with Mettler Toledo TGA/SDTA system (Greifensee, Switzerland). Briefly, 5–8 mg of lyophilized sample was loaded in a platinum pan and its energy level was scanned in the ranges of 30–480°C and 30–450°C respectively under a nitrogen atmosphere, with a temperature gradient of 10°C min^-1^. Analysis was performed under gradual increase in temperature, plotting the weight percentage and heat flow against temperature respectively.

### Study of BS stability

Stability studies were performed using the cell free broth obtained by centrifugation at 8000 × g for 20 min. 10 ml of BS suspension (25 mg ml^-1^) was kept at 0, 5, 15, 25, 50, 75, 100, and 125°C for 30 min, cooled to room temperature; the surface tension and emulsification index were determined. To elucidate the pH stability of BS, the sample was adjusted to different values (5.0-12.0) with 1 M NaOH and 1 M HCl, and the same aforesaid measurements were performed. All the assay were carried out in triplicates.

### Biofilm inhibition assay on silicone tubes

The biofilm forming pathogenic strains of *Escherichia coli* ATCC 25922, *Staphylococcus aureus* ATCC 6358P, *Pseudomonas aeruginosa* ATCC 15442*, Bacillus cereus* ATCC 11770*, Listeria monocytogenes* MTCC 1143 and *C. albicans* MTCC 183 were used as indicators in the biofilm inhibition assay on silicone tubes. Briefly, 10 μl volumes of overnight bacterial cultures were added into 1000 μl of fresh Luria broth (LB) medium. The same volume of *C. albicans* was also added into 1000 μl of fresh YPD medium. 1000 μl cell-bound partially purified BS (50 mg ml^-1^) was added (final concentration of BS in solution was 25 mg ml^-1^) in LB and YPD medium. Furthermore, 4 cm long pieces of sterile silicone tubes (Himedia, India) incubated with medium amended with BS for 24 h at 37°C. After incubation, silicone tubes were removed and washed twice with distilled water. Air dried silicone tubes were further stained with 0.1% crystal violet solution for 20 min. The stained silicone tubes were washed twice with distilled water and allowed to dry at room temperature for 30 min (Janek et al. 2013).

### Pre-adhesion treatment on polystyrene surface with BS

The antiadhesive activity of the crude BS derived from isolate MRTL 9 against several biofilm forming pathogen strains was quantified according to the procedure described by Heinemann *et al.* (2000). Briefly, the wells of a sterile 96 well flat bottomed plastic tissue culture plate (Himedia, India) were filled with 200 μl of the crude biosurfactant. The plate was incubated for 18 h at 4°C and subsequently washed twice with PBS. Control wells contained PBS buffer only. An aliquot of 200 μl of a washed bacterial or yeast suspension was added and incubated in the wells for 4 h at 4°C. Unattached microbial cells were removed by washing the wells three times with PBS. The adherent cells were fixed with 200 μl of methanol (Himedia, India) per well. Then the plates were stained for 5 min with 200 μl of 2% crystal violet solution. Excess stain was washed out by placing the plate under running tap water. The dye bound to the adherent micro-organisms was resolubilized with 200 μl of 33% (v/v) glacial acetic acid (Himedia, India) and the absorbance of each well was measured at 600 nm. The inhibition percentages at different biosurfactant concentrations for each microorganism were calculated as:



Where Ac represents the absorbance of the well with a biosurfactant concentration c and A_0_ the absorbance of the control well.

### Cytotoxicity assessment

The cytotoxicity of BS was evaluated on mouse fibroblast (ATCC L929) cell line (Cochis et al. 2012). The cells were cultured in Dulbecco Modified Eagle Medium (DMEM) at 37°C in 5% CO_2_ atmosphere. A standardized quantity of cells (1 × 10^4^) was inoculated in 100 μl of DMEM in 96 well culture plates and incubated for stabilization for 24 h before the treatment. The stock solution of BS was prepared in DMSO (99.9%) at concentration of 10 μg/1 μl. The final quantities of BS was added 25 μg, 12.5 μg and 6.25 μg and incubated for 24 h at 37°C in 5% CO_2_ atmosphere. After 24 h, 15 μl dye solution from the Cell Titre 96® non-radioactivity cell proliferation assay kit (Promega, USA) was added in to the wells and kept for 4 h incubation as per the recommendation. 100 μl stopping solution was added in all the wells and incubated overnight to dissolve formazan product to get uniform readings. The absorbance was recorded at 570 nm in microplate spectrophotometer (SPECTRAmax, Molecular devices, USA). The DMSO used as solvent was taken as negative control in the assay (amount equal to prepare BS dilution). To estimate the cytotoxicity of biosurfactant, microbially originated Rhamnolipid (25 mg ml^-1^) and Sodium dodecyl sulfate (25 mg ml^-1^) were used as positive and negative controls respectively. All the data is analyzed and plotted in advance graphic software Slide Write Plus™ v.6.

## Results and discussion

### Screening for BS production

Initially the LAB isolate was screened for ability to produce surface active agents. The selection of BS producing strain was a two stages selection process. Primarily the isolate was screened for their ability to produce BS using drop collapse assay. The flattened drop of PBS containing BS over the paraffin liquid coated surface confirmed the presence of BS. In addition to the surface activity, efficient emulsification property is critical for promising BS and its applications (Banat et al. 2000). Probiotic strain of *L. acidophilus* was isolated and screened for surface active agents using drop collapse assay (Tahmourespour et al. 2011). The emulsification ability of BS was assayed and maximum emulsification activity was found with Kerosene oil (E_24_ = 64%). Drop collapse method and emulsification index are semi-quantified methods, so in need to quantify significant reduction in ST was measured by Tensiometer. The potential of a microbial surfactant is determined by its capability to reduce the surface tension of production medium. A potent BS can decrease the ST of distill water from 72.0 to 35.0 mN m^-1^ (Busscher et al. 1994; Mulligan 2005).

The BS produced by strain MRTL9 showed a significant reduction in ST of PBS extract from 72 to 40.2 mN m^-1^. Various LAB strains were reported as BS producer on the basis of their ability to reduce surface tension of production media. A significant reduction in surface tension was reported while working with different strains of *L. casei* and *Bifidobacterium* from 72 to 35.5 mN m^-1^ (Goŀek et al. 2009). Whereas, *Streptococcus thermophilus* A and *Lactococcus lactis* 53 reduced surface tension around 36.0-37.0 mN m^-1^ (Rodrigues et al. 2004). In addition to the ST, CMC of the purified BS was found to be approximately 2.25 mg ml^-1^ in Tris–HCl buffer, pH 8. The flattened drop collapse, significant ST reduction and potent emulsification index decisively confirmed the biosurfactant production by the strain.

### Taxonomic identification

Isolate MRTL 9 was taxonomically identified using 16S rRNA sequencing using universal primers. Genomic DNA was directly amplified by colony PCR using universal 16S rRNA primers. Taxonomic affiliation of the isolates was retrieved from GenBank. The blast algorithm (http://blast.ncbi.nlm.nih.gov/Blast.cgi) was used to determine the most related sequence relatives in the GenBank database. Isolate was identified as *Enterococcus faecium* after sequence alignment using BLAST algorithms*.* Nucleotide sequences has been deposited in the NCBI GenBank database (http://www.ncbi.nlm.nih.gov/) with the accession number KC456369. Incidence of *E. faecium* were also reported earlier from various fermented foods with beneficiary probiotic attributes (Albesharat et al. 2011; Grosu-Tudor et al. 2013).

### Production and purification of BS

**Figure 1:**
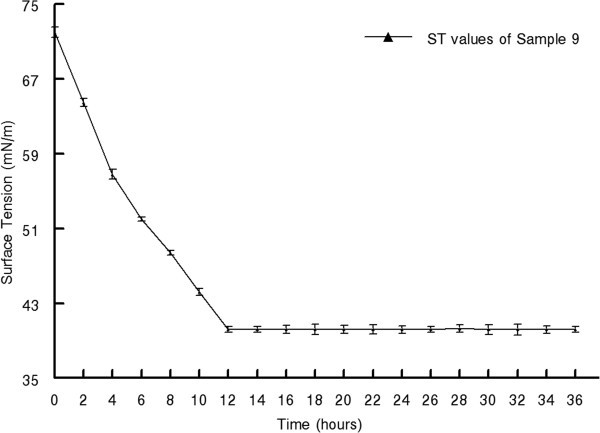
**Decrease of surface tension with time by cell bound BS.**

The production of BS was carried out by inoculating with 1% overnight pre culture at 37°C in a rotary shaker for 72 h. After incubation, ST of the cell bound BS in PBS reduced to 40.2 mN m^-1^ from 72 mN m^-1^. ST of the production media was reduced with the course of incubation time (Figure 1). The significant decrease of ST established the production of BS by the *E. faecium* MRTL 9. Maximal ST reduction was observed at late logarithmic and early stationary phase of cell growth. Reduction in ST during the logarithmic and stationary phase has been reported and confirmed the production of BS (Oliveira and Garcia-Cruz 2013; Madhu and Prapulla 2014). BS was extracted twice with equal volume of ethyl acetate; crude BS (brown in color) was obtained and partially purified using column chromatography (Silica gel; 60–120 mesh size). Fraction with highest activity were pooled and analyzed by different thin layer chromatography (TLC) with post chromatographic detections.

### Characterization of BS

TLC is the most extensively studied method for structural determination of BS (Carrillo et al. 1996; Mohebali et al. 2007). BS was initially characterized by TLC followed by post chromatographic detection. Developed silica plate when sprayed with Syldatk reagent produced a dark red spot indicating the presence of sugar moieties. Further, replica plate was sprayed with ammonium molybedate produced gives a dark blue spot indicated the presence of lipid moiety. No amino acid residues were detected while stained with Ninhydrin reagent. The above results of TLC characterization confirmed the presence of the glycolipid BS. Generally glycolipids are carbohydrates in amalgamation with long-chain aliphatic or hydroxy aliphatic acids (Lang 2002). A glycolipid like moiety was reported extremely surface active, reducing the surface tension of water to 35 mN m^-1^ (Velraeds et al. 1997; Sauvageau et al. 2012). Partially purified cell bound BS was appeared as crystalline dirty white powder. The BS formed white precipitation line between sample well and cationic compounds CTAB with barium chloride. The BS derived from *E. faecium* was confirmed as an anionic BS. Generally BS produced from other LAB were found as anionic surfactants. Xylolipid produced by *Lactococcus lactis* was also reported anionic in nature (Saravanakumari and Mani 2010).

### FTIR analysis

**Figure 2:**
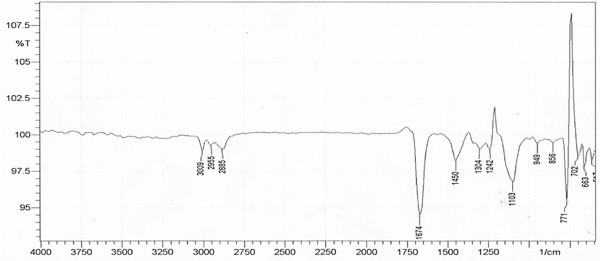
**FTIR spectra of BS produced by** ***E. faecium*** **MRTL9.**

Several reports have been published on BS produced by LAB but inadequate information about their chemical composition is known. LAB derived BS were initially characterized as multicomponent mixtures consisting of protein fractions, polysaccharides and phosphate groups (Velraeds et al. 1996; Rodrigues et al. 2004; Thavasi et al. 2008; Falagas and Makris 2009; Sauvageau et al. 2012; Schippers et al. 2000). BS derived from *E. faecium* MRTL 9 in stationary growth phase was biochemically characterized as glycolipid (Figure 2, Table 1). Analysis of FTIR spectrum of BS sample revealed the composition as lipid and polysaccharide fractions. The molecular composition of BS revealed that most prominent adsorption bands were located at 3009 cm^-1^, 2855 cm^-1^ and 2955 cm^-1^ (C–H stretching bands of CH_2_ and CH_3_ groups), 1674 cm^-1^ (C = O stretching vibrations of the carbonyl groups), 1103 cm^-1^ (C–O stretching bands; formed between carbon atoms and hydroxyl groups in the chemical structures) and 771 cm^-1^ (CH_2_ group) which prominently confirmed the presence of glycolipid type of BS (Thavasi et al. 2008 and Rodrigues et al. 2006b). According to FTIR spectrum obtained, the BS is composed of carbohydrates and lipid. The hydrophobic chain of BS is composed of lipid and hydrophilic part is mainly composed of sugar. Comparison of the spectra obtained revealed that the BS is closely similar to Xylolipid (glycolipid) reported earlier from different LAB strains (Thavasi et al. 2008, 2011; Saravanakumari and Mani 2010).

**Table 1:** **Absorbance maxima of IR spectra and functional groups detected in the BS produced by** ***Enterococcus faecium***

Absorbance range (cm^-1^)	Functional groups detected
3000–3600	OH stretching, typical polysaccharides
2900–2950	C-H (stretching) groups CH_2_ and CH_3_
1725, 1675	C = O (stretching of carbonyl group)
1400–1460	C = H stretching
1000–1300	C-O sugar stretching
675-1000	C-H bending

### NMR spectroscopy

**Figure 3:**
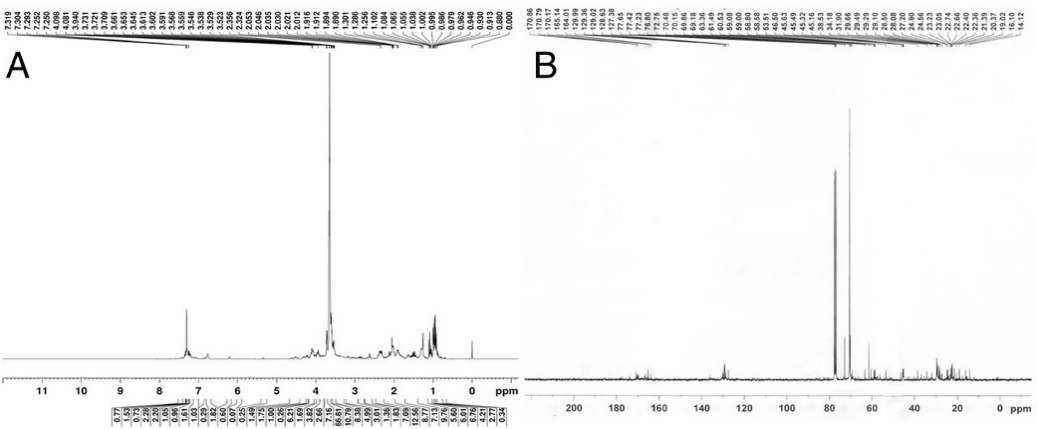
^**1**^ **H NMR (A) and** ^**13**^ **C NMR (B) spectrum of BS produced by** ***Enterococcus faecium*** **MRTL9.**

Furthermore, the spatial arrangement of proton and carbon in bio-molecule has been carried out by ^1^H NMR and ^13^C NMR spectroscopy. The typical chemical shifts were pointed out that the BS has the molecular structure closely resembles to glycolipid (Figure 3, Table 2). The chemical shifts were harmonized with those of previous reports of glycolipids. A number of peaks were observed in NMR due to the presence of sugar moieties and aliphatic fatty acids chains. The proton NMR spectra seems complex, however some distinguished peaks were present and their chemical shift was evaluated. The presence of chemical shift (δ) at 4.09 ppm, 3.53 ppm, 3.53 ppm, 2.35 ppm, 3.72 ppm and 3.52 ppm indicated C_1_, C_2_, C_3_, C_4_, C_5_, OCH_3_ and –COOH groups respectively in proton NMR spectra. The presence of chemical shifts at 127.38 ppm, 60 ppm, 69.5 ppm, 58.81 ppm, 53.51 ppm and 170.79 ppm were also depicted C_1_, C_2_, C_3_, C_4_, C_5_, OCH_3_ and –COOH groups respectively in carbon NMR spectra. Characteristic spectra peaks of NMR were also reported in Xylolipid from *Lactococcus lactis* (Saravanakumari and Mani 2010) and other closely related glycolipids (Henkel et al. 2012; Morita et al. 2012; Lotfabad et al. 2013; Desai and Banat 1997; Falagas and Makris 2009).

**Table 2:** **Chemical shift assignment (NMR data) of BS produced by** ***Enterococcus faecium*** **MRTL9**

Assignment(s)	^1^H NMR (ppm)	^13^C NMR (ppm)
C-1	4.09	127.38
C-2	3.53	~60
C-3	3.53	~60
C-4	2.35	69.5
C-5	3.72, 3.61	58.81
OCH_3_	3.52	53.51
COOH	-	170.79

### UPLC MS analysis

The molecular mass of the active compound was measured using UPLC/ESI-MS. A prominent peak with a retention time of 25.29% was observed. The liquid chromatography coupled with mass spectrum confirmed the presence of glycolipid type of BS possibly composed of lipid and polysaccharide fractions. The possible predicted structure of separated product is close and similar to the glycolipid with hexadecanoic fatty acid chain.

### GC-MS analysis of fatty acids of methyl esters (FAME)

**Figure 4:**
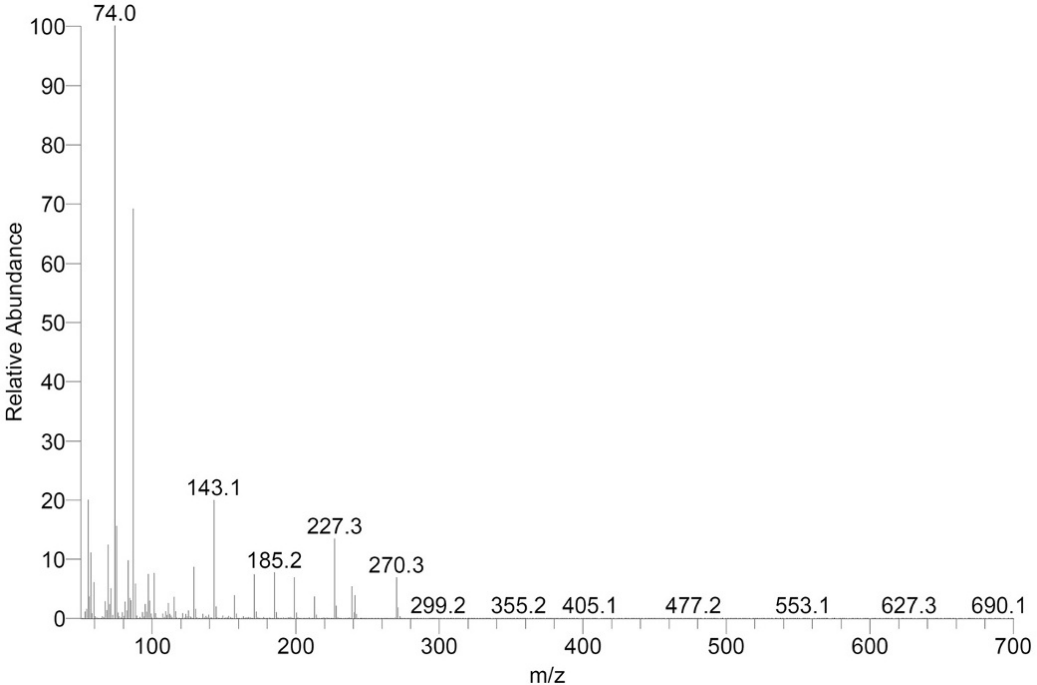
**GCMS separation of BS produced by the** ***E. faecium*** **MRTL9 mainly showing peaks for hexadecanoic acid.**

**Figure 5:**
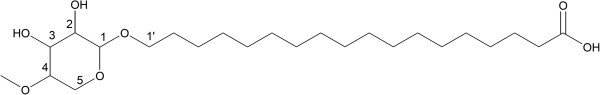
**Composite structure of biosurfactant produced by** ***E. faecium*** **MRTL9 (Xylolipid).**

The fatty acid composition of was BS analyzed by GC-MS and compared with the library data. It was found that BS mainly comprised of long chain fatty acids, mainly C-16 long fatty acids (Figure 4). The major fatty acid of BS produced was C-16 hexadecanoic fatty acid (86.6%). Hexadecanoic acid was found as main fatty acid chains in various studies of glycolipids purified previously. Saravanakumari and Mani (2010) have isolated BS from *L. lactis* which also contains octadecanoic acid as a fatty acid chain associated with sugar moiety. Rhamnolipids are the extensively isolated glycolipids which are also composed of β-hydroxydecanoic acid molecules as branched fatty acids (Desai and Banat 1997). Palmitic acid and stearic acid were found major fatty acid type in cell bound biosurfactant produced by *L. pentosus* (Vecino et al. 2014). On the basis of FTIR, NMR (Proton and carbon) liquid and gas chromatography the structure of BS produced by *E. faecium* elucidated and predicted as Xylolipid with hydrophillic part as xylose sugar and hydrophobic part as β-hydroxydecanoic acid (Figure 5). There is one carboxyl group connected with the sugar and aliphatic hydrocarbon chain. The carboxyl group provided overall negative charge on the biomolecule which could be useful for binding and thus remediating divalent cations (Gutierrez et al. 2009)

### Thermal gravimetric analysis (TGA)

**Figure 6:**
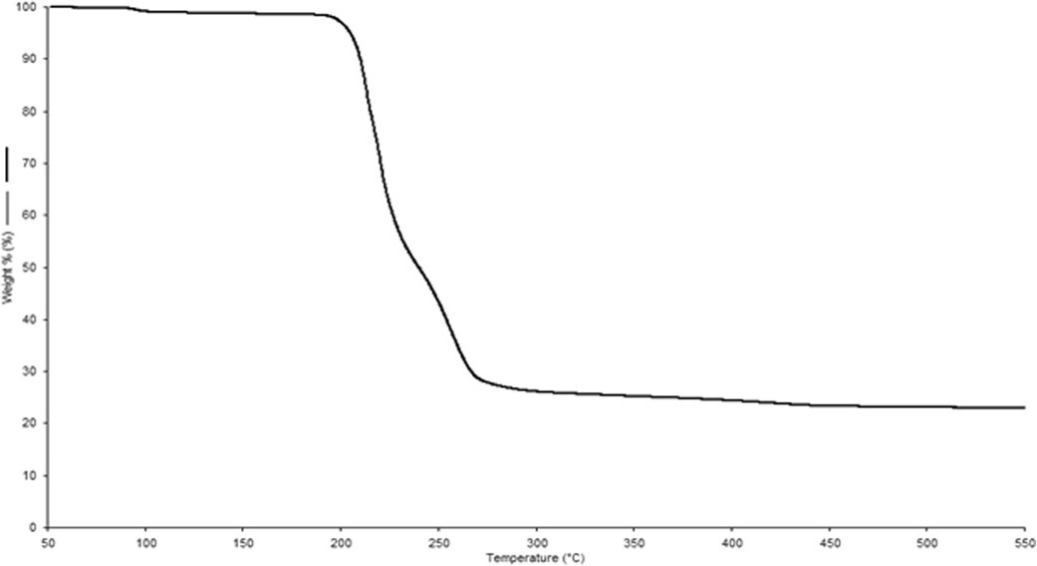
**Thermal degradation analysis of BS produced by the** ***E. faecium*** **MRTL9.**

Thermal stability of BS is a significant property for its commercial application at elevated ranges of temperature such as microbial enhanced oil recovery and food industries. Thermal degradation of BS was conducted by TG analysis. Approximately (1%) of weight loss was observed with increase in initial temperature from 50 to 200°C possibly due to the loss of solvents and moisture molecules. Complete loss of BS was observed after 270°C. Moisture release during heating of the polymer suggested that the polymer was not truly anhydrous. The degradation temperature (Td) was found to be 250°C determined from TGA curve (Figure 6). It was previously documented that the BS produced from alkalophilic strain of *Klebsiella* spp. showed maximum degradation at 350–400°C (Jain et al. 2013). Similar findings were also reported while working on the rhamnolipid produced by *Pseudomonas aeruginosa* MA01. The weight of polymer was dramatically lost around 290°C and continued gradually to decrease (Abbasi et al. 2012). BS isolated from the strain *E. faecium* showed similar thermal degradation behavior close to the glycolipids. BS produced by *E. faecium* was found to be thermostable. As molecular mass determined by mass spectroscopy confirmed that the BS isolated in the present study having similar molecular mass close to the glycolipid BS and also showed similar thermal degradation behavior.

### Stability of BS

**Figure 7:**
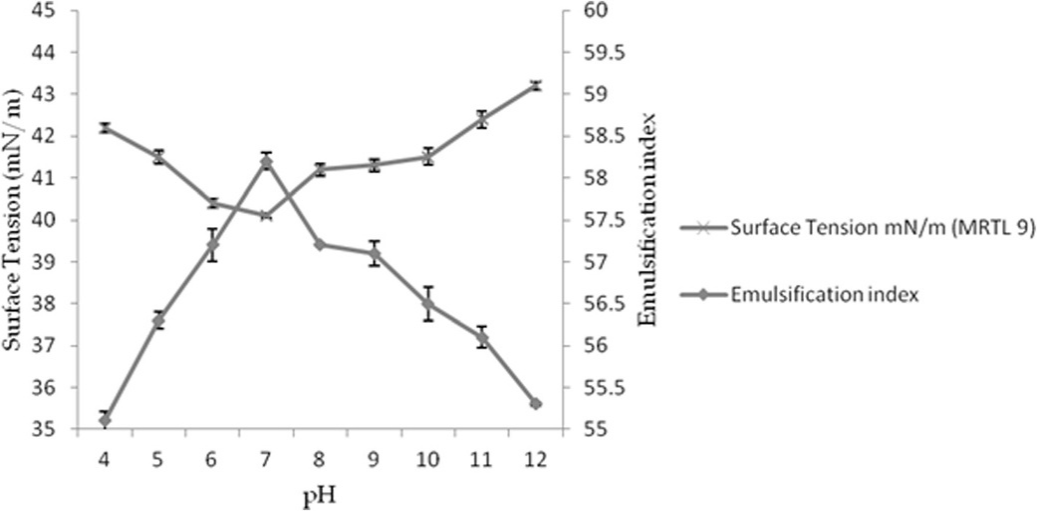
**Effect of pH on the surface tension of the cell-bound partially purified BS isolated from** ***E. faecium*** **MRTL9.**

**Figure 8:**
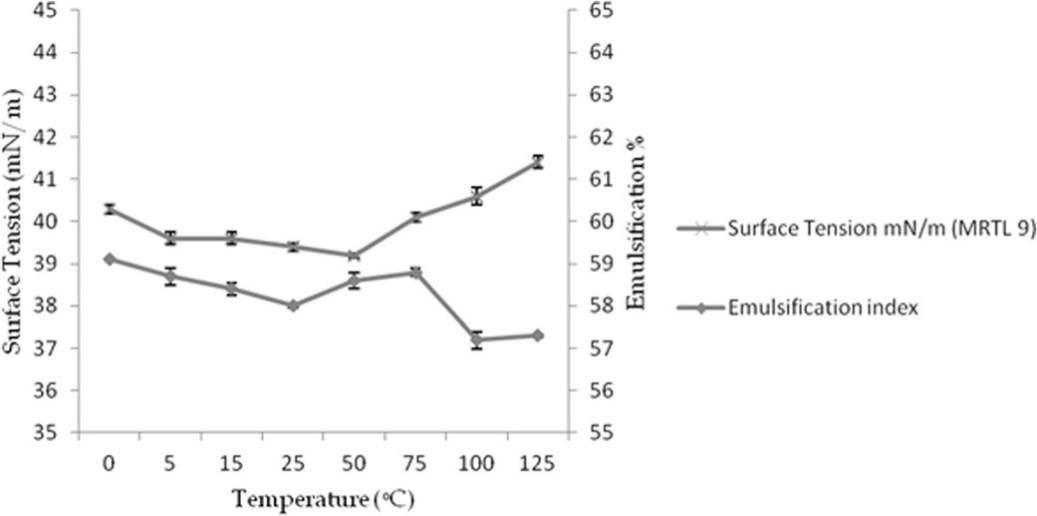
**Effect of temperature on the surface tension of the cell-bound partially purified BS isolated from** ***E. faecium*** **MRTL9.**

The application of BS is depends on their stability at extreme temperatures and pH range. The stability of BS was studied at different pH, temperature and observed in conviction of effect on ST and emulsification index. Figures 7 and 8, present the effects of temperature and pH on surface tension and emulsification capacity of cell bound BS. In current study, partially purified BS retained activity over pH range from 6 to 10 with a minimal deviation in surface tension values. The pH values below and upper 7 showed minor increase in surface tension but found to be stable, the BS sustained activity even after treatment at 0–120°C for 30 min. The effect of different pH and incubation at extreme temperature on surface tension and emulsification capacity were insignificant. Heat treatment (autoclaving at 120°C for 15 min) on BS caused no appreciable changes in their surface and emulsifying activities (Desai and Banat 1997). Biosurfactant produced by *Pseudomonas aeruginosa* SP4 was also found stable isolated from petroleum contaminated soil (Pornsunthorntawee et al. 2008). BS from *Candida lipolytica* cultivated with industrial residue and a lipopeptides from *Bacillus subtilis* C9 cultivated with a carbohydrate substrate demonstrated similar stability behavior (Rufino et al. 2008). Lipopeptides of *Bacillus subtilis* also reported with stability at elevated temperature and different range of pH (Vaz et al. 2012). The BS was structurally stable and retained its surface activity even at the extreme pH and high temperature range. Stability of BS at high temperature also conferred in accordance with the results obtained after TG analysis.

### Biofilm removal on silicone tubes

**Figure 9:**
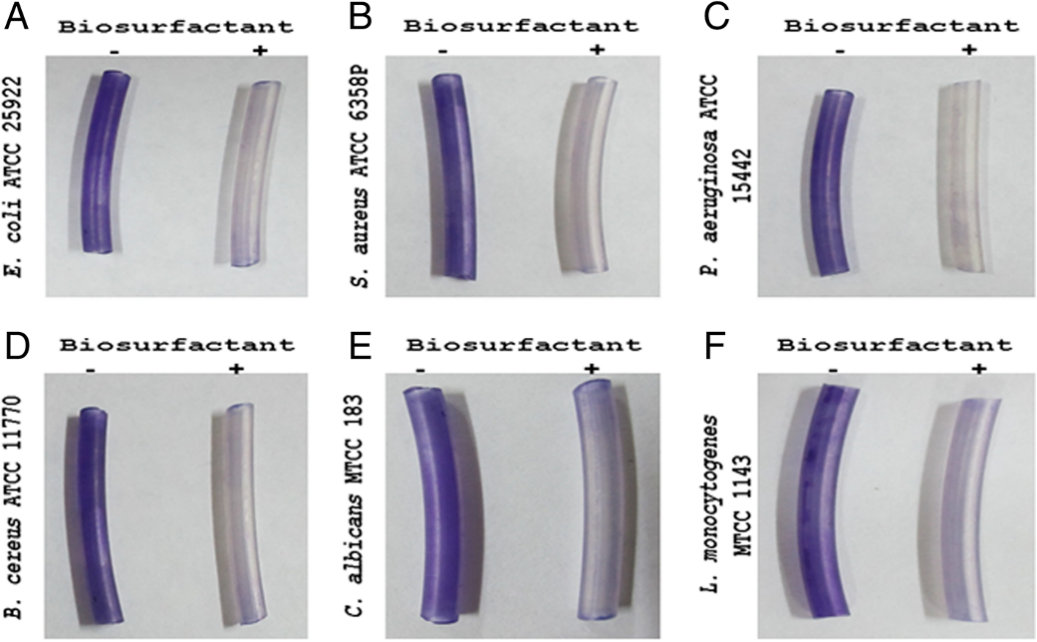
**BS produced by** ***E. faecium*** **inhibits biofilm formation of (A)** ***E. coli*** **ATCC 25922 (B)** ***S. aureus*** **ATCC 6358P (C)** ***P. aeruginosa*** **ATCC 15442 (D)** ***B. cereus*** **ATCC 11770 (E)** ***C. albicans*** **MTCC183 (F)** ***L. monocytogenes*** **MTCC1143 on silicone tubes, (-) without BS treatment (+) with BS coating at the concentration of 25 mg ml** ^**-1**^ **.** Biofilm inhibition was visualized by staining with crystal violet.

The biofilm developed by all the pathogenic strains was spread along the whole tube surface (Figure 9). Biofilm formation reduced significantly on silicone tube even at a low concentration of BS (25 mg ml^-1^). The pre incubation of silicone tubing with known concentration of BS was effective against all biofilm forming pathogens. Moderate anti-biofilm activity was observed against the yeast pathogenic strain *C. albicans.*

Falagas and Makris (2009) has proposed the application of BS isolated from probiotic LAB to patient care equipment’s in hospitals to combat colonization by microorganisms responsible for nosocomial infections. BS derived from *S. thermophiles* A was found to be effective antiadhesive substance on silicon rubber and combat colonization of various microorganisms (Rodrigues et al. 2006). The number of bacterial cells adhering to the silicone rubber with preadsorbed BS reduced by 89% and 97% by two strains after 4 h incubation. BS with a concentration of 25 mg ml^-1^, was able to reduce *S. aureus*, *S. epidermidis* and *S. agalactiae* population significantly on silicone rubber tubing. Application of BS derived from *L. paracasei* subsp. *paracasei* A20 was found to be a potent antiadhesive molecule for elimination of fungal and bacterial pathogens on polystyrene surface (Gudina et al. 2011). BS was also found effective in prevention of colonization of yeast on medical surfaces. Anti-adhesive property of BS derived from *Lactobacillus* sp. against *Candida albicans* in pre-coating assay was able to reduced 82% at concentration of 312.5 μg/ml (Fracchia et al. 2010).

BS produced by *E. faecium* MRTL 9 strain was found to reduced microbial population from silicone tubing near to completely. BS showed anti-adhesive properties against commonly known biofilm formers on silicone tubing of medical equipment’s and surgical implants. More than 95% reduction was achieved with 25 mg ml^-1^ of BS added to the growth medium. BS incorporated surface cleaning agents could be a next generation material for operational theater and highly sophisticated lab surfaces. The present study established a platform onwards in developing alternative strategies to prevent microbial colonization on surgical implants and silicone rubber prostheses.

### Biofilm removal on polystyrene surface

The antibiofilm property of BS was evaluated against various pathogenic strains (Table 3). The BS showed antibiofilm activity against most of the pathogens but the spectrum of activity was varied for different microorganisms and also depends upon the concentration of the BS used. The highest antibiofilm property was observed against *L. monocytogenes* (97.1%), *E. coli* (95.4%) *B. cereus* (91.2%), *P. aeruginosa* (89.6%) *S. aureus* (83.1%) and least antiadhesive potential was observed against *C. albicans* (65.3%) strain. The antibiofilm activity against *C. albicans* was quite low even at the highest biosurfactant concentration assayed *i.e.* 25 mg ml^-1^. Biosurfactant produced by *E. faecium* MRTL 9 strain reduced biofilm on polystyrene surface near to completely. Contribution of biosurfactant in microbial desorption has been widely observed, and application of biosurfactant of lactobacilli to solid surfaces might be an effective approach to reduce microbial population of pathogenic strains (Rodrigues et al. 2006). The results of present experimental setup suggests the possible use of the BS of *E. faecium* as an alternative to conventional antimicrobial agents for bio-medical applications.

**Table 3:** **Removal of biofilm on polystyrene surfaces by biosurfactant isolated from** ***E. faecium*** **at different range of concentration (mg ml** ^**-1**^ **)**

Test organisms	Biosurfactant (mg ml^-1^)					Control
	25	12.5	6.25	3.12	1.56	PBS
*E. coli*	95.4 ± 0.65	82.1 ± 0.39	74.1 ± 0.15	51 ± 0.11	40.9 ± 0.36	0
*P. aeruginosa*	89.6 ± 0.52	69.2 ± 0.25	55.1 ± 0.11	44.1 ± 0.16	39.2 ± 0.46	0
*S. aureus*	83.1 ± 0.47	73.3 ± 0.38	69.3 ± 0.05	55.3 ± 0.30	39. ± 0.14	0
*L. monocytogenes*	97.1 ± 0.38	84.3 ± 0.38	63.1 ± 0.17	49.3 ± 0.32	32.4 ± 0.51	0
*B. cereus*	91.2 ± 0.16	79.3 ± 0.28	61.1 ± 0.28	39.2 ± 0.20	28.1 ± 0.20	0
*Candida albicans*	65.3 ± 0.16	63.0 ± 0.51	44.1 ± 0.17	30.2 ± 0.20	19.4 ± 0.45	0

### Cytotoxicity assessment

**Figure 10:**
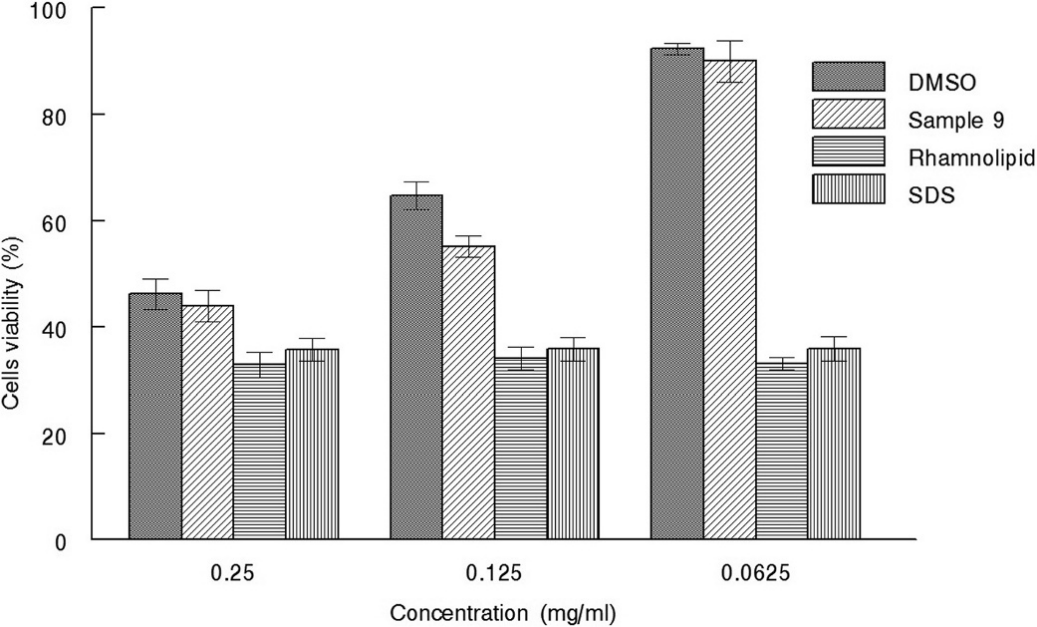
**Cytotoxic effect of BS produced on mouse fibroblast cell at different concentration.**

Nowadays there is an immense concern regarding the toxicity and safety of BS used for therapeutic purposes. So, there is an instant need to spot safety of BS. Cytotoxicity of BS was evaluated using mouse fibroblast (ATCC L929) cell line. The Mouse fibroblasts cells were selected and generally regarded suitable for cytotoxicity assessment. Mouse fibroblast cells are recommended for in vitro evaluation of medical devices by the International Organization for Standardization (2009). During cytotoxicity determination different concentrations of cell bound BS and purified rhamnolipids (Janeil, USA) were prepared in DMSO (Figure 10). Whereas SDS at equal concentration was used as negative control. Significant differences in cell viability of mouse fibroblasts cell was observed at concentration of 25 mg ml^-1^, 12.5 mg ml^-1^ and 6.25 mg ml^-1^. Cell viability was found maximum about 90% at 6.25 mg ml^-1^ in case of BS produced by strain MRTL9 while positive control rhamnolipid showed 35.33% viability quiet close to SDS *i.e.* 35%. But increase in the concentration of BS also declined the cellular viability. At concentration of 25 mg ml^-1^, cell viability was found 43.93% as compared to rhamnolipid which showed 32.87% of cell viability. While DMSO used as diluent did not showed any significant cytotoxicity. A total of 92.27% cell viability was observed at 6.25 mg ml^-1^. The outcomes of cellular toxicity showed lower toxicity of BS in comparison to SDS and rhamnolipid. SDS has been admired as a reference irritant because of its fast-acting, non-allergenic, and toxicity (Effendy and Howard 1996).

Various reports on evaluation of cytotoxic evaluation of biosurfactant came in literature, the lack of cytotoxicity is anticipated when you wish to ecofriendly and safe antiadhesive suspension directly to be used for human applications and food applications. Typically, the cytotoxicity seems linked to its interactions with the phospholipids of cell membrane and therefore cell lysis. Cochis et al. (2012) has reported biosurfactants cytotoxicity on mouse fibroblast cell line with concentrations ranges from 25 to 6.25 μg ml^-1^. Pre-coating with BS caused a greater reduction in biofilm cell number and cell viability than chlorhexidine. BS produced by *Sphingobacterium detergens* was studied for its cytotoxicity and antiproliferative effects in different cell lines. When comparing cytotoxicity values (IC_50_) of the two fractions in fibroblast and keratinocyte cell cultures, Fraction B was found less cytotoxic, showing lower toxicity than the reference compound SDS, indicating low skin irritability (Burgos-Díaz et al. 2013; Kumar et al. 2014). According to the outcomes of present study, BS produced by *E. faecium* interferes with microbial adhesion and demonstrate cyco-compatibility with mouse fibroblasts.

## Conclusion

The BS produced by *E. faecium* MRTL 9 was isolated and structurally characterized as similar to Xylolipid. Structurally the BS characterized as a glycolipid with hexadecanoic fatty acid (C16) chain. In addition, biosurfactant was confirmed as non-cytotoxic compound as compared with other microbial and chemically synthesized surfactant. BS obtained from the LAB strain was found to be stable at different pH and also at elevated temperature ranges. Furthermore, due to its significant antiadhesive property and non-cytotoxic nature, the BS can potentially be used as a cleaning/coating material for bio-medical equipment’s. The results obtained in this study regarding the antiadhesive properties of this lactobacilli derived BS also opens new future prospects for its use for combating microbial colonization, making it a suitable alternative to conventional antimicrobials.
